# Abnormal Activity Detection Using Pyroelectric Infrared Sensors

**DOI:** 10.3390/s16060822

**Published:** 2016-06-03

**Authors:** Xiaomu Luo, Huoyuan Tan, Qiuju Guan, Tong Liu, Hankz Hankui Zhuo, Baihua Shen

**Affiliations:** 1 School of Medical Information Engineering, Guangzhou University of Chinese Medicine, Guangzhou 510006, China; tanhuoyuan@gmail.com; 2 College of Mechanical and Electrical Engineering, Zhongkai University of Agriculture Engineering, Guangzhou 5102256, China; qiuju95@126.com; 3 Department of Electronic Science, Huizhou University, Huizhou 516007, China; liutongboss@163.com; 4 School of Data and Computer Science, Sun Yat-sen University, Guangzhou 510006, China; zhuohank@mail.sysu.edu.cn; 5 School of Information Engineering, Guangdong University of Technology, Guangzhou 510006, China; bhshen@sohu.com

**Keywords:** pyroelectric infrared (PIR) sensor, abnormal activity detection, wireless sensor network

## Abstract

Healthy aging is one of the most important social issues. In this paper, we propose a method for abnormal activity detection without any manual labeling of the training samples. By leveraging the Field of View (FOV) modulation, the spatio-temporal characteristic of human activity is encoded into low-dimension data stream generated by the ceiling-mounted Pyroelectric Infrared (PIR) sensors. The similarity between normal training samples are measured based on Kullback-Leibler (KL) divergence of each pair of them. The natural clustering of normal activities is discovered through a self-tuning spectral clustering algorithm with unsupervised model selection on the eigenvectors of a modified similarity matrix. Hidden Markov Models (HMMs) are employed to model each cluster of normal activities and form feature vectors. One-Class Support Vector Machines (OSVMs) are used to profile the normal activities and detect abnormal activities. To validate the efficacy of our method, we conducted experiments in real indoor environments. The encouraging results show that our method is able to detect abnormal activities given only the normal training samples, which aims to avoid the laborious and inconsistent data labeling process.

## 1. Introduction

The world population is aging rapidly. As time goes on, the proportion of the elderly relative to the total population has increased, and continues to increase, especially in developed countries [1]. Thus, helping seniors live a better life is crucial and has great societal benefits. Although the elders have the option of going to nursing homes or hospice care, most of them would prefer to stay in their own houses where they feel more familiar and comfortable. Limited funding for public healthcare services and the shortage of registered nurses are also driving factors for the adoption of a home-based assisted living paradigm. Therefore, healthy aging at home has become one of the most active research areas [1], especially the problem of abnormal activity detection [2,3,4,5]. The elderly living alone in isolated areas have been in need of emergency attention, and in the worst cases, some were found dead in their homes [6].

Traditionally, abnormal activity detection approaches use cameras to obtain the data of full human body movements [7]. However, there are challenging issues in vision-based methods, such as computational complexity in image processing, data consistency under different illumination conditions, and privacy infringement of the human target [8]. These problems make the practical deployment of vision-based systems difficult. An alternative method is to collect sensing data from wearable motion sensors and detect abnormal activities based on the collected sensing data [2]. Although motion sensors worn on the human body or integrated into human clothing can collect motion data with much less volume of data compared to those from vision-based systems, such wearable devices may make the human subject feel obtrusive. In addition, the elders are prone to forget wearing the devices after they change clothes. In addition, having to recharge the wearable devices regularly, even after deliberate design of power management units, is inconvenient for the user [2]. In order to serve as a reliable and robust abnormal activity detection system for the elderly living alone, the following factors should be considered:

1. robust to the change of environment, especially the light illumination;
2. protective to the residents’ privacy;
3. convenient to use, especially for the elderly.

Bearing those factors in mind, a Pyroelectric Infrared (PIR) sensing paradigm offers a promising alternative to the optical and wearable counterparts [3]. PIR sensors are non-intrusive sensors and only sensitive to the infrared radiation changes induced by human motion, which makes them robust to interference caused by clustered background and illumination variance. In addition, as PIR sensors are relatively cheap and can be embedded within home environments, such as ceiling-mounted deployment, they are suitable to be used for home-based assisted living.

However, there are some challenges facing the PIR based sensing system for abnormal behavior detection. The first one is the design of sensor nodes, which are required to capture the spatio-temporal characteristic of the human motion. Furthermore, as the data generated continuously by ambient PIR sensors, there is an increasing demand to analyze those ever-growing sensing data automatically, with little human intervention. Most importantly, the problem of abnormal activity detection is computationally challenging [4]. Here, we define abnormal activities as events that they have not been expected in advance. Unlike normal activities, the abnormal samples are extremely scarce, or even non-existent. It is impossible to acquire or simulate all kinds of abnormal samples to train the system beforehand.

In this paper, we propose a PIR-based sensing system for anomaly detection. We design a PIR sensor node that can capture the spatio-temporal feature of human motion effectively. The key to achieve this target is by leveraging the visibility modulation of each sensor to enhance spatial resolution. We employ the reference structure tomography (RST) paradigm [9] to segment the Field of View (FOV) of each PIR sensor into sampling cells. Thus, different human activities will generate discriminative spatio-temporal signals under the monitor region. Next, we use the Hidden Markov Models (HMMs) [10], one kind of generative models, to profile normal activities. Each training sample is modeled by an HMM, and their dissimilarity is calculated based on the Kullback–Leibler (KL) divergence [11]. A self-tuning spectral clustering algorithm is used to cluster similar training samples, without the need to specify the number of cluster and the distance kernel width manually [12]. Finally, One-Class Support Vector Machines (OSVM) [13] are setup to profile the normal activities, and any unexpected activities will be classified as anomaly. It is worth pointing out that our system is trained in an unsupervised manner, which aims to avoid the laborious and inconsistent manual data labeling process.

The rest of the paper is structured as follows: Section 2 introduces the related work. Section 3 provides the design and implement of the PIR sensors. Section 4 offers the overview of our proposed method. Section 5 presents the framework of human activity representation, dissimilarity calculation and clustering. Section 6 depicts the usage of the OSVM algorithm for abnormal activity detection. The experimental results are provided in Section 7. Conclusions and future work are given in Section 8.

## 2. Related Works

Traditionally, cameras have been used for human activity classification and abnormal activity detection [14]. The processing of video includes background subtraction, human motion extraction and activity modeling [15]. The video data streams usually contain tens of thousands of pixels in each frame, and their intensity is easily effected by the change of illumination [5]. The excessive computational burden and the feeling of privacy intrusion make it difficult to be employed massively in real home environment.

Wearable sensors is another paradigm for anomaly detection. Yin *et al.* [4] propose a two-stage approach for detecting abnormal activities. The first stage is to train an OSVM to model the commonly normal activities, and the second stage is to derive abnormal activity models from the suspicious activities filtered by the first stage. Zhu *et al.* [2] propose using the wearable sensors together with location information provided by the camera to detect abnormal activities. A probabilistic framework is used to model different anomalies, including spatial anomalies, timing anomalies, duration anomalies and sequence anomalies. However, the biggest inconvenience of wearable sensing is that the sensors have to be recharged regularly even after deliberate design of power control units [2].

PIR sensing is a promising choice besides camera-based and wearable-based sensing. In [16], three PIR sensor models which are deployed in a hallway are used to detect the movement of eight human targets, including the two moving directions, three distance intervals and three speed levels. PIR sensor models can also be used to construct wireless sensor networks, which are intended to track and recognize multiple human targets [17]. Using only the binary information obtained by infrared sensors attached to the ceiling of a room, the human positions can be estimated, and even the number of humans in the room changes dynamically [18].

To capture the spatio-temporal feature of human movement, the monitored region is segmented into discrete sampling cells [19]. By leveraging the idea of compressive infrared sampling, the FOV of each PIR sensor is modulated by reference structure [9]. In [20], PIR sensors were used to extract the spatio-temporal feature of the human motion from the infrared radiation domain. Ten aerobic exercises, 360 examples in total, which were performed in front of the PIR sensor node were recorded, and the nearest neighbor classifier was then used to classify different exercises. Furthermore, they presented a PIR-based compressive classification method for recognizing six typical physical activities [21]. Similar to the experimental setup in [16], three sensor models were located on the ceiling, with opposite tripods facing each other. SVM and HMMs were used to evaluate the performance of their system [21].

PIR sensors are also used to detect abnormal activities, especially fall detection. In [22], PIR sensors were deployed in a distributed sensing paradigm, which aimed at capturing the synergistic motion patterns of head, upper-limb and lower-limb. The experiment results of fall detection were encouraging. However, their system was side-view, which means it was easily occluded by other objects, and the falls had to occur perpendicular to the FOV of the PIR sensors. In other words, it was view-dependent. To overcome these limitations, Luo *et al.* [3] proposed using the ceiling-mounted PIR sensor array to implement a fall detection system, *SensFall*. To achieve fall detection, the normal and abnormal training samples had to be collected beforehand. In other words, it was the supervised machine learning paradigm [23]. However, the abnormal detection is clearly a cost sensitive problem [4] because the samples of abnormal behavior were rare, or even non-existent. All types of the anomalies can not be elaborated on in advance. If we can only acquire the sensing data generated from normal activities, how can we train the system for abnormal activity detection automatically? This is the motivation of our study.

In this paper, we propose a PIR sensor based sensing paradigm for abnormal activity detection in an unsupervised fashion. To avoid the laborious and inconsistent manual data labeling process, we propose using the self-tuning spectral clustering algorithm to discover the number of normal activities automatically. The KL divergence is employed to measure the similarity between each pair of normal training samples and construct the similarity matrix. HMMs are then utilized to profile each cluster of training samples, and OSVM is trained to detect abnormal activities. The details of our method will be elaborated on in the following sections.

## 3. Sensing System

### 3.1. Sensing Model

In this subsection, we review the design of our sensing model. The task of the sensing model is to capture the discriminative spatio-temporal feature of the human activities.

**Figure 1:**
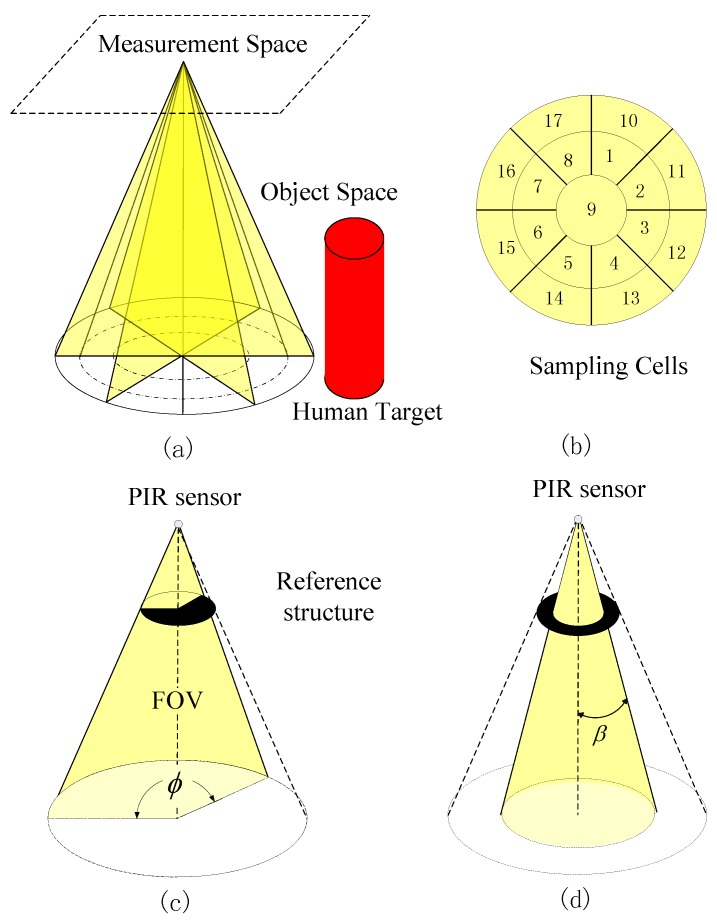
Sensing model design: (**a**) Measurement space, object space and the human thermal target; (**b**) The projection of sampling cells on the ground; (**c**) Type I mask; (**d**) Type II mask.

The schematic diagram of our sensing model is shown in Figure 1a. Our model springs from the reference structure tomography (RST), which uses multidimensional modulations to encode the mapping between radiating objects and sensor measurements [9]. The object space refers to the space where humans perform different activities. It is the 3D physical space where human motion will generate varying radiation patterns. The measurement space refers to the space where the PIR sensors are located. Before visibility modulation, the output of all the PIR sensors are the same; they can not be used for activity classification. To capture the spatio-temporal feature of human motion, we segment the object space into discrete cells. The projection of these sampling cells on the ground is shown in Figure 1b. Assume that the object space Ω is divided into *L* discrete non-overlapping sampling cells, denoted as $\Omega_{i}$, then $\Omega =\cup_{i}\Omega_{i},\Omega_{i}\cap \Omega_{j}=\emptyset$, where 1≤i,j≤L.

Assume that there are *M* sensors located in the measurement space. The visibility function $v_{ji}$ is binary valued, depending on whether the sampling cell $\Omega_{i}$ is visible to the *j*th PIR sensor:

$$\begin{array}{c} v_{ji}=\left\{\begin{array}{cc} 1 & \;\;\Omega_{i}\;\mathrm{is}\;\mathrm{visible}\;\mathrm{to}\;\mathrm{the}\;j\mathrm{th}\;\mathrm{PIR}\\ 0 & \;\;\mathrm{otherwise} \end{array}\right. \end{array}$$

The output of the *j*th PIR sensor is given by

(1) $$\begin{array}{ccc} m_{j}\left(t\right) & = & h\left(t\right)*\sum_{i=1}^{L}v_{ji}\int_{\Omega_{i}}s\left(r,t\right)dr\\ & = & \sum_{i=1}^{L}v_{ji}\left[h\left(t\right)*\int_{\Omega_{i}}s\left(r,t\right)dr\right]\\ & = & \sum_{i=1}^{L}v_{ji}s_{i}\left(t\right) \end{array}$$

where “*” denotes convolution, h(t) is the impulse response of the PIR sensor, and $\Omega_{i}\in R^{3}$ is the *i*th sampling cell. s(r,t) is the thermal density function in the object space, and then $s_{i}\left(t\right)=h\left(t\right)*\int_{\Omega_{i}}s\left(r,t\right)dr$ is the sensor measurement of sampling cell $\Omega_{i}$.

Equation (1) can be equivalently represented in a matrix form as

(2) $$\begin{array}{c} M=VS \end{array}$$

where $M=\left[m_{j}\left(t\right)\right]\in R^{M\times 1}$ is the measurement vector of PIR sensors, $V=\left[v_{ji}\right]\in R^{M\times L}$ is the measure matrix determined by the visibility modulation scheme, and $S=\left[s_{i}\left(t\right)\right]\in R^{L\times 1}$ is the sensor measurement of the sampling cells before visibility modulation. M can be regarded as a linear measurement of the radiation variation within all cells.

The human body can be regarded as an infrared radiative source to the surrounding environment. In comparison with the whole object space, the human body is sparsely distributed. Thus, the change of infrared radiation induced by human motion takes place only in a few sampling cells. This can be regarded as a compressive sensing problem [24], and the activity classification in the object space is projected into an analogous problem in the measurement space. When the signal is sparse or compressible, learning and classification directly in the compressive measurement domain are possible in the compressed sensing framework [21,25].

### 3.2. Reference Structure Implementation

To implement the sensing model described in the previous subsection, which segments the object space into discrete sampling cells, we employ two kinds of masks. These masks play the role of reference structure, which modulates the FOV of PIR sensors. The first type of mask, Type I, is a fan shape, as shown in Figure 1c. After applying such mask, the FOV of the PIR sensor is no longer a full cone, but a partial cone shape, called a fan cone. The second type of mask, Type II, is a ring shape, as shown in Figure 1d. The FOV of the PIR sensor after masked is still a full cone, but its cone angle *β* is less than that of the original cone. These two types of masks provide two degrees of freedom (DOF) spatial partitions.

**Figure 2:**
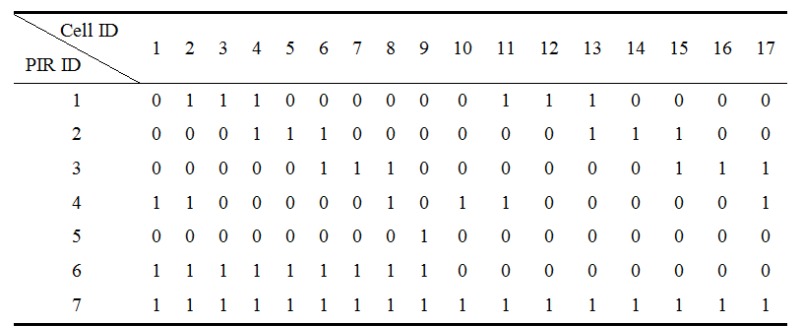
Measurement matrix: seven pyroelectric infrared (PIR) sensors for 17 sampling cells.

In our system implementation, the performance of our system will improve as the number of PIR sensors increases [20]. Because of the hardware constraint of our sensor node, seven PIR sensors with masks are multiplexing to segment the object space into sampling cells, as shown in Figure 1b. Four PIR sensors are masked by Type I mask, and the remaining three PIR sensors are masked by the Type II mask. In such a configuration, the object space is segmented into 17 sampling cells. Referring to Equation (2), M=7,L=17, and the measurement matrix *V* is shown in Figure 2.

## 4. Proposed Algorithm

**Figure 3:**
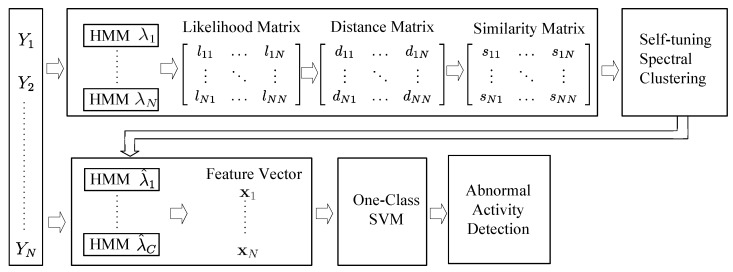
A block diagram illustrating our approach.

Based on the implementation of our sensing model, human activity under the object space will generate PIR data streams correspondingly. The measurement of PIR sensors are segmented by the Short-Time Energy method automatically [3,26]. Given a collection of normal samples $\left\{Y_{1},Y_{2},...,Y_{N}\right\}$, our abnormal activity detection method works in two phases. Figure 3 shows a diagrammatic illustration of our method. In the first phase, by applying the self-tuning spectral clustering, the number of activities classes *C* is determined automatically, and the normal traces are grouped accordingly. In the second phase, each class of activity is modeled by an HMM. The equal-length feature vectors are constructed based on the likelihood output of training samples generated by these *C* HMMs. The OSVM is then trained for abnormal activity detection. It shows clearly that the spectral clustering algorithm is the core of our approach. The key components of our approach are explained in detail in the following sections.

## 5. Spectral Clustering

To profile the normal activities in an unsupervised fashion, we employ the spectral clustering method to cluster similar sequences. However, because the lengths of these sequential data are different and vary greatly in value, it is a challenging issue to model these data for better similarity measures.

### 5.1. Likelihood Matrix Construction

Since the training sequences are generated by a hidden mechanism associated with human’s underlying activities, it is reasonable to model these sequences using a generative model [27]. In this work, we adopt a set of HMMs to model the training sequences. HMMs are a type of non-deterministic stochastic finite state automata, which are widely employed in signal processing and pattern recognition [28]. The parameters of a continuous HMM with Gaussian mixture emissions can be represented in the following compact form:

(3) $$\begin{array}{c} \lambda =\{\pi ,A,\mu ,\Sigma \} \end{array}$$

where *π* is the initial state probability distribution, *A* is the state transition probability distribution, *μ* is the mean vector, and Σ is the covariance matrix.

The *i*th training sequence $Y_{i}$ can be presented as the output of *M* PIR sensors,

(4) $$\begin{array}{c} Y_{i}=\left[\begin{array}{cccc} m_{1}\left(1\right) & m_{1}\left(2\right) & \ldots & m_{1}\left(T_{i}\right)\\ \vdots & \vdots & \ddots & \vdots \\ m_{M}\left(1\right) & m_{M}\left(2\right) & \ldots & m_{M}\left(T_{i}\right) \end{array}\right] \end{array}$$

where $m_{j}\left(t\right)$ is the output of the *j*th PIR sensor at time *t*, $t=1...T_{i}$. By using the Baum–Welch algorithm [10], we fit *N* HMMs, one for each individual sequence $Y_{i},1\leq i\leq N$.

To calculate the distance between each pair of these sequences, a probabilistic model-based framework for sequence clustering is proposed in [29]. The likelihood matrix $L=\{l_{ij}\}$, whose ijth element is defined as

(5) $$\begin{array}{c} l_{ij}=\log\;p_{ij}=\frac{1}{\mathrm{length}(Y_{j})}\log P\left(Y_{j};\lambda_{i}\right),1\leq i,j\leq N \end{array}$$

where $Y_{j}$ is the *j*th sequence, $\lambda_{i}$ is the model trained for the *i*th sequence, and $P(Y_{j};\lambda_{i})$ is the likelihood of $Y_{j}$ generated by model $\lambda_{i}$.

### 5.2. Sequence Distance Measures

After the likelihood matrix L is constructed, the original variable-length sequence clustering problem is transformed to a typical similarity-based one. The *j*th column of L represents the likelihood of sequence $Y_{j}$ under each of the trained models. The next step is to define a meaningful distance measure for these sequences.

A popular paradigm is to obtain likelihood-based distances between each pair of sequences [29]. Based on this work, several other distance measures have been proposed under a similar philosophy [27,30,31]. However, the main limitation of these methods is that they only consider the distance between two sequences each time, not including the global information of the whole set of data. Hence, we propose using the definition of distance measurement from a probabilistic perspective [32].

We regard the likelihood of each of the sequences under the trained models as samples from the conditional likelihoods of the models given the data, which embeds information from the whole data set [32]. This gives rise to highly structured distance matrices to give a better performance in comparison with aforementioned distance-based methods [27,29,30,31].

According to the definition of likelihood matrix L, the *j*th column of L can be regarded as the likelihood of the sequence $Y_{j}$ under each of the trained models $\lambda_{i},1\leq i\leq N$. These *N* models can be regarded as a set of “sampled points” from the model space Λ surrounding the HMMs that actually span the data space. Thus, these *N* trained models become a good discrete approximation $\bar{\Lambda }=\left\{\lambda_{1},...,\lambda_{N}\right\}$ to the model space of interest.

If we normalize the likelihood matrix L, which means each column adds up to one, we get a new matrix $L_{N}$ whose columns can be regarded as the probability density functions (pdfs) over the approximated model space conditioned on each of the individual sequences:

(6) $$\begin{array}{c} L_{N}=\left[f_{\bar{\Lambda }}\left(Y_{1}\right),...,f_{\bar{\Lambda }}\left(Y_{N}\right)\right] \end{array}$$

This interpretation leads to the Kullback–Leibler (KL) divergence, which is a natural choice for the measurement of the dissimilarity between two pdfs. The discrete case of the KL divergence formulation is as follows:

(7) $$\begin{array}{c} D_{KL}(f_{P}\left|\right|f_{Q})=\sum_{i}f_{P}\left(i\right)\log\frac{f_{P}\left(i\right)}{f_{Q}\left(i\right)} \end{array}$$

where $f_{P}$ and $f_{Q}$ are two discrete pdfs. Obviously, the KL divergence is not a proper distance because of its asymmetry; a symmetrized version is used as:

(8) $$\begin{array}{c} D_{KL}^{sym}(f_{P}\left|\right|f_{Q})=\frac{1}{2}[D_{KL}(f_{P}\left|\right|f_{Q})+D_{KL}(f_{Q}\left|\right|f_{P})] \end{array}$$

Thus, the distance between the sequences $Y_{i}$ and $Y_{j}$ can be defined as:

(9) $$\begin{array}{c} d_{ij}=D_{KL}^{sym}(f_{\bar{\Lambda }}\left(Y_{i}\right)\left|\right|f_{\bar{\Lambda }}\left(Y_{j}\right)) \end{array}$$

Distances defined this way are obtained according to the patterns created by each sequence in the probability space spanned by different models, and the distance measured between two sequences $Y_{i}$ and $Y_{j}$ involves information related to the rest of the data sequences.

### 5.3. Similarity Matrix Construction

Before applying a spectral clustering algorithm, the distance matrix $D=\{d_{ij}\}$ should be transformed into a similarity matrix $S=\{s_{ij}\}$. A commonly used procedure is to apply a Gaussian kernel,

(10) $$\begin{array}{c} s_{ij}=\left\{\begin{array}{cc} exp(-\frac{d_{ij}^{2}}{2\sigma^{2}}) & \mathrm{for}\;i\neq j\\ 0 & \mathrm{for}\;i=j \end{array}\right. \end{array}$$

where *σ* is the scaling parameter controlling the kernel width.

The value of *σ* is commonly specified manually, or numerous iteration has to be run for a range of *σ* [33]. However, when the input data includes clusters with different local statistics, a single value of *σ* may not work well for all the data. Thus, instead of selecting a single scaling parameter, we propose calculating a local scaling $\sigma_{i}$ for each data point $d_{i}$ [12]. The similarity between $Y_{i}$ to $Y_{j}$ can be revised as $d_{ij}/\sigma_{i}$ while the converse is $d_{ji}/\sigma_{j}$. Therefore, $d_{ij}$ is symmetry, and the Equation (10) can be generalized as:

(11) $$\begin{array}{c} {\hat{s}}_{ij}=\left\{\begin{array}{cc} exp(-\frac{d_{ij}^{2}}{\sigma_{i}\sigma_{j}}) & \mathrm{for}\;i\neq j\\ 0 & \mathrm{for}\;i=j \end{array}\right. \end{array}$$

where $\sigma_{i}=d\left(Y_{i},Y_{K}\right)$, $Y_{K}$ is the K’th neighbor of $Y_{i}$. The selection of *K* is independent of scale and is a function of the data dimension of the embedding space.

Thus, the scaling parameters for each pair of $Y_{i}$ and $Y_{j}$ are not fixed; they are determined automatically according to the local statistics of the neighborhoods.

### 5.4. Self-Tuning Spectral Clustering

After the similarity matrix $\hat{S}=\left\{{\hat{s}}_{ij}\right\}$ is constructed, we apply spectral clustering methods to partition the training sequences into clusters. For an undirected graph *G* with vertices $v_{i}$ and edges $s_{ij}$, the matrix S could be considered as an adjacent matrix for *G*, where each element $s_{ij}$ can be viewed as the similarity between the vector $v_{i}$ and $v_{j}$. The target of spectral clustering is to partition the *G* into a distinct sub-graph.

It is a tricky problem to specify the number of clusters *C*. One method to discover the number of clusters is to analyze the eigenvalues of the normalized Laplacian matrix $L_{a}$, which is based on the similarity matrix $\hat{S}$. The analysis given in [33] shows the number of repeated eigenvalues of magnitude 0 with multiplicity equal to the number of clusters *C*. However, eigenvalues depend on the structure of the individual clusters, and no assumptions can be placed on their values [12]. Once noise is introduced, the eigenvalues deviate from the ideal case, and it is difficult to decide the number of clusters.

An alternative approach to discover the number of clusters *C* automatically is to analyze the eigenvectors of Laplacian matrix $L_{a}$ [12]. Assume the matrix $X=\left[x1,...,x_{C}\right]\in R^{N\times C}$ is constructed by stacking the largest eigenvectors of $L_{a}$ in columns. In the ideal case where the data points could be separated distinctly, X will be strictly block diagonal after sorting the eigenvectors of $L_{a}$. Nevertheless, in the general case, the X’s off-diagonal blocks are non-zero, and the eigensolver could just pick any other set of the orthogonal vectors; X could have been replaced by $\hat{X}=\mathrm{XR}$ for any orthogonal matrix $R\in R^{C\times C}$. Now, we have to recover the rotation which best aligns X’s columns with the canonical system with the minimum cost.

Let $Z\in R^{N\times C}$ be the matrix obtained after rotating the eigenvector matrix X; that is, Z=XR. We wish to recover the rotation R for which, in every row in Z, there will be at most one non-zero entry. We thus define the cost function:

(12) $$\begin{array}{c} J=\sum_{i=1}^{N}\sum_{j=1}^{C}\frac{Z_{ij}^{2}}{M_{i}^{2}} \end{array}$$

where $M_{i}=\max_{j}Z_{ij}$. Minimizing this cost function over all possible rotations will provide the best alignment with the canonical coordinate system. The number of clusters, *C*, is taken as the one providing the minimal cost.

The spectral clustering algorithm that we apply is similar to the one proposed in [12]. The algorithm works as follows:

1. Define a diagonal degree matrix $D=\{d_{ij}\}$ with $d_{ii}=\sum_{j=1}^{N}{\hat{s}}_{ij}$, and then construct the normalized Laplacian matrix $L_{a}=D^{-1/2}\hat{S}D^{-1/2}$.
2. Find $C^{\prime }$ principal eigenvectors $x_{1},x_{2},...,x_{C^{\prime }}$ and form the matrix $X=\left[x1,...,x_{C}^{\prime }\right]\in R^{N\times C^{\prime }}$ by stacking the eigenvectors in columns, where $C^{\prime }$ is the largest possible cluster number.
3. Recover the rotation R which best aligns X’s columns with the canonical coordinate system using the incremental gradient descent algorithm [12].
4. According to Equation (12), grade the cost of the alignment for each group number up to $C^{\prime }$. Set the final group number $C_{\text{best}}$ to be the largest group number with minimal alignment cost.
5. Take the alignment result Z of the $C_{\text{best}}$ eigenvectors, and assign the original point $s_{i}$ to cluster c if and only if ${\text{max}}_{j}\left(Z_{ij}^{2}\right)=Z_{ic}^{2}$.

In our experiments, because the voulunteers will emulate five kinds of activities, $C^{\prime }$ is set to 10, and the self-tuning spectral clustering will determine $C_{\text{best}}$ automatically.

## 6. One-Class SVM Classifier

### 6.1. Feature Extraction

After applying the spectral clustering algorithm, we can group the *N* training traces into *C* clusters, which correspond to *C* different types of activities.

To train an OSVM, we need to transform the training samples that are of variable lengths into a set of fixed-length feature vectors. Again, we apply HMMs to model these normal activities, one for each cluster. For each learned model with the corresponding parameters ${\hat{\lambda }}_{i}$, 1≤i≤C, we calculate the log-likelihood of each of the *N* normal traces given the model parameters ${\hat{\lambda }}_{i}$. The log-likelihood value for each pair of trace and HMMs is computed as follows:

(13) $$\begin{array}{c} L\left(Y_{i};{\hat{\lambda }}_{j}\right)=\log P\left(Y_{i};{\hat{\lambda }}_{j}\right),1\leq i\leq N,1\leq j\leq C \end{array}$$

This is calculated by applying the standard forward-backward algorithm [10]. In this way, for each training trace $Y_{i},1\leq i\leq N$, we can obtain a *C*-dimensional feature vector $x_{i}=\langle L\left(Y_{i};{\hat{\lambda }}_{1}\right),...,L\left(Y_{i};{\hat{\lambda }}_{C}\right)\rangle$.

### 6.2. One-Class SVM Training

After transforming the *N* training traces into a set of feature vector $x_{1},...,x_{N}$, we can train the one-class SVM for normal activities. The basic idea is to find a sphere that contains most of the normal data such that the corresponding radius *R* can be minimized:

(14) $$\begin{array}{cc} \min_{R,\xi ,a} & \;R^{2}+\nu \sum_{i=1}^{n}\xi_{i}\\ s.t. & \;||x_{i}-a{\left|\right|}^{2}\leq R^{2}+\xi_{i}\\ & \;\xi_{i}\geq 0 \end{array}$$

The slack variables $\xi_{i}$ are introduced to allow some data points to lie outside the sphere, and the parameter ν≥0 controls the tradeoff between the volume of the sphere and the number of errors. Using the dual representation of the Lagrangian [34], the objection function is equivalent to

(15) $$\begin{array}{cc} \min_{\alpha } & \sum_{i,j=1}^{n}\alpha_{i}\alpha_{j}\langle x_{i}\cdot x_{j}\rangle -\;\sum_{i=1}^{n}\alpha_{i}\langle x_{i}\cdot x_{i}\rangle \\ s.t. & \;0\leq \alpha_{i}\leq \nu ,\sum_{i=1}^{n}\alpha_{i}=1 \end{array}$$

This quadratic programming (QP) problem can be solved using standard optimization techniques [35]. To determine if a testing sample is within the sphere, the distance to the center of the sphere has to be calculated. If the distance is larger than the radius *R*, the testing sample is considered abnormal.

Typically, the training samples are not spherically distributed in the input space. Thus, the original data points are first mapped into a feature space so that a better data description can be obtained. Instead of requiring an explicit mapping function from the input space to the feature space, the solution can be obtained by replacing all the inner products 〈·,·,〉 in Equation (15) by a kernel function k(·,·):

(16) $$\begin{array}{c} \min_{\alpha }\;k\left(x_{i}\cdot x_{j}\right)-\sum_{i=1}^{n}\alpha_{i}k\left(x_{i},x_{i}\right) \end{array}$$

In our context, due to the noisy and nonlinear characteristic of the PIR sensors, the decision boundary of the OSVM is quite complex. Thus, we apply the Gaussian Radial Basis Function (RBF) kernel for the OSVM, which is defined as follows:

(17) $$\begin{array}{c} k\left(x_{i},x_{j}\right)=exp(-\gamma ||x_{i}-x_{j}{\left|\right|}^{2}) \end{array}$$

where γ>0 is a scaling factor that controls the width of the kernel function.

## 7. Experimental Evaluation

In order to evaluate the performance of our proposed method, experiments were carried out on a real data set collected from a wireless sensor network. Our proposed method is referred to as SC+OSVM in the experiments. Two other approaches were also used for comparison. We list these three methods as the following:

- SC+OSVM—The method proposed in our study, mainly including self-turning spectral clustering and One-Class SVM.
- SC+iForest—The difference between this method and the SC+OSVM is that we use isolation forest to replace One-Class SVM for abnormal detection. Isolation forest is an alternative algorithm for abnormal detection [36].
- OneHMM—All the normal training samples are modeled by only one HMM, which corresponds to not applying spectral clustering to the unlabeled samples. A threshold is set to distinguish normal and abnormal activities.

### 7.1. Experimental Setup

**Figure 4:**
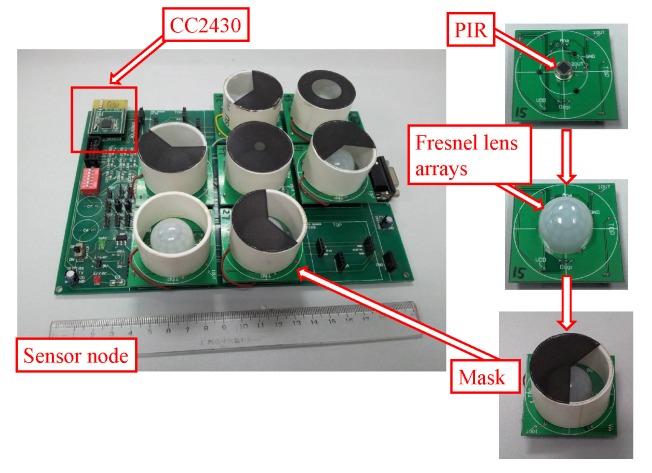
The wireless sensor node: there are seven PIR sensors on one sensor node. The sensor node is mounted at a height of 3m from the floor, looking down to classify human activities. Each PIR sensor is equipped with its own fresnel lens arrays and mask. The sampling frequency of PIR sensor is 25 Hz, and the resolution of the A/D converter is 8-bit. The CC2430 is the Radio Frequency (RF) transmission module based on ZigBee protocol, the transmission rate of which is 250 Kbps at 2.4 GHz.

The experiments were carried out in a real indoor environment. The monitored region covered by the sensor node was a cone with a 3 m radius. The sensor node is with seven PIR sensors, each of them equipped with fresnel lens arrays and a mask, as shown in Figure 4. The CC2430 module is used for data transmission between the sensor node and the sink based on ZigBee protocol. More details of the sensor node could be found in our previous work [3].

**Figure 5:**
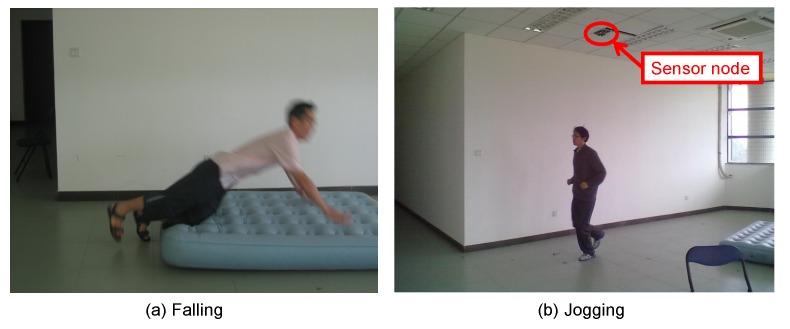
Different volunteers emulated different activities with their own speed and strategy: (**a**) Falling; (**b**) Jogging.

There were eight volunteers that participated in our experiments, including three females and five males. The height of them ranges from 1.64 m to 1.80 m, and the weight of them ranges from 50 kg to 70 kg. Each volunteer emulated five kinds of activities, including falling, sitting down, standing up from a chair, walking and jogging. Every activity was emulated ten times by each volunteer at a self-select speed and strategy, as shown in Figure 5. Totally, we obtained 400 samples, including 80 fall-simulated samples and 320 normal activity samples. In our experiments, fall-simulated samples are regarded as abnormal activities, and other samples are regarded as normal activities. Each sample is segmented automatically by thresholding Short-Time Energy, and all the normal training samples are unlabeled.

### 7.2. Evaluation Metrics

The performance of the abnormal human-activity detection methods can be evaluated in terms of two rates, detection rate and false alarm rate. The detection rate is computed as the ratio of the number of correctly detected abnormal activities to the total number of abnormal activities. The false alarm rate is computed as the ration of the number of normal activities that are incorrectly detected as abnormal activities to the total number of normal activities.

Based on the confusion matrix shown in Table 1, the two metrics can be defined as follows:

(18) $$\begin{array}{cc} \text{Detection}\;\text{Rate} & =\frac{TP}{TP+FN} \end{array}$$

(19) $$\begin{array}{cc} \text{False}\;\text{Alarm}\;\text{Rate} & =\frac{FP}{FP+TN} \end{array}$$

The performance of an ideal abnormal human-activity detection algorithm should have a high detection rate and a low false alarm rate. Therefore, we evaluate the performance of the algorithms using an Receiver Operating Characteristic (ROC) curve, which plots the detection rate against the false alarm rate. In addition, we compute the area under the ROC curve (AUC) to compare these algorithms. AUC is a better measurement than accuracy in the evaluation of learning algorithms [37,38], especially in the cost-sensitive problems. A desirable algorithm with a high detection rate and a low false alarm rate should have an AUC value closer to one.

### 7.3. Experimental Results

**Figure 6:**
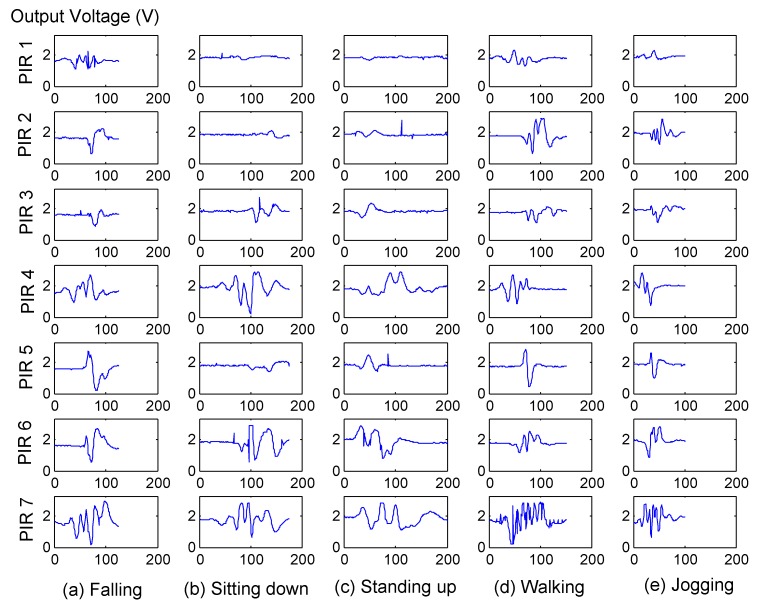
The original output of the sensor node with seven PIR sensors for different activities: (**a**) Falling; (**b**) Sitting down; (**c**) Standing up; (**d**) Walking; (**e**) Jogging. The horizontal axis represents the sampling points (25 Hz) of the activity.

In our experiments, because the output of the sensor node was a seven-dimensional data stream with continuous values, as shown in Figure 6, we employed HMMs with Gaussian observation density. The number of hidden states and the number of Gaussian models are determined by the log-likelihood of the training samples [3]. Specifically, we use HMMs with two Gaussians and eight states to profile the general human normal activity and each cluster of normal activities. For OSVM, the parameter *ν* was set to 0.01.

**Figure 7:**
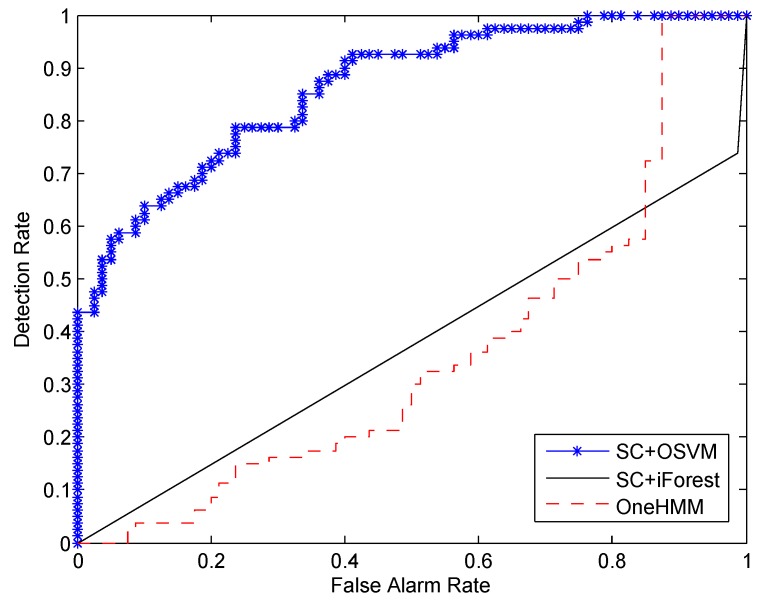
Comparison of the detection rate and the false alarm rate *versus* different numbers of training samples: training on 240 normal samples.

Experiments were conducted to compare the performance of all three of the algorithms. We randomly selected 240 normal samples for training. The other 80 normal samples and all the 80 abnormal samples were randomly mixed together for testing. Figure 7 shows the ROC curve with respect to the detection rate and the false alarm rate. We can see from the figure that SC+OSVM gives the best detection result. This is mainly because the self-turning spectral clustering can estimate the number of different types of activities automatically and then cluster the similar activities accordingly. The discriminative feature vectors are helpful to improve the accuracy of OSVM.

**Figure 8:**
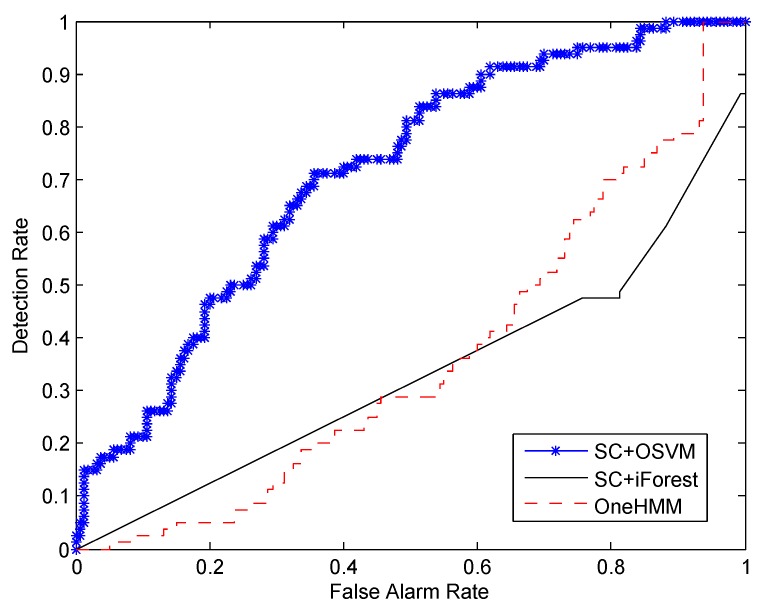
Comparison of the detection rate and the false alarm rate *versus* different numbers of training samples: training on 160 normal samples.

**Figure 9:**
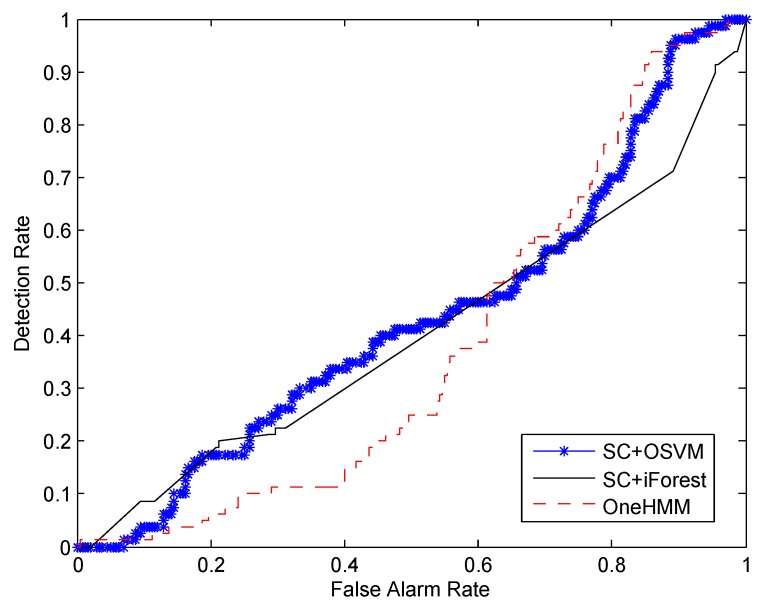
Comparison of the detection rate and the false alarm rate *versus* different numbers of training samples: training on 80 normal samples.

We also conducted experiments to investigate the effect of varying the number of training samples on the performance of the three algorithms. In these experiments, we kept the amount of testing data unchanged and reduced the number of training samples. Figure 8 and Figure 9 show the experimental results using 160 and 80 normal traces for training, respectively. We can see from the figure that, when the number of normal traces for training decreases, the performance of the three algorithms decreases correspondingly. The reason lies in two aspects. First, for HMMs, which are generative models [3,9], sparse training samples affect the accuracy of parameter estimation of the models. Second, for OSVM, when the training samples are sparse, the calculated decision boundary may not exactly capture the characteristics of normal activities. Therefore, the abilities of three algorithms to distinguish normal and abnormal activities degrade. As shown in Figure 9, when there are only 80 normal samples for training, the performance of OneHMM and SC+iForest are comparable to that of SC+OSVM because all of them could not model the normal activities well with less training samples. When the training samples are less than 80, the performance of three algorithms will be worse and unsatisfactory. Again, as shown in Figure 8, when we have 160 normal samples for training, SC+OSVM still performs the best among the three algorithms.

To explicitly compare the performance of the three algorithms, we computed the AUC values by calculating the area under the ROC curves depicted in Figure 7, Figure 8 and Figure 9. The results are summarized in Table 2. We can see from the first column of the table that, when 240 normal samples are used for training, the AUC value for SC+OSVM, SC+iForest and OneHMM are 0.863, 0.375 and 0.354, respectively. SC+OSVM performs better than the other two algorithms. In addition, SC+OSVM is the best among the tree algorithms when we consider 80 and 160 normal samples for training as well.

Another observation is that the performance of OneHMM will not be improved as the number of training samples increases. This is because the dissimilarity between different activities will degrade the classification ability of the HMMs; a single HMM to model all normal activities is not discriminative enough. By contrast, after applying the self-turning spectral clustering, each type of normal activities will be profiled by an HMM; it will obviously improve the performance of the OSVM.

## 8. Conclusions

In this paper, we propose a novel approach for detecting a human’s abnormal activities. By employing the FOV modulation for PIR sensors, the human activity is encoded into low-dimensional data streams, which can be used to extract the tempo-spatial feature of the human motion. A self-turning spectral clustering algorithm is used to cluster the training samples with modified KL distance. The HMMs are trained to profile each cluster of activity. One-class SVM is setup to classify whether the testing samples are abnormal or not. A major advantage of our approach is that it does not need abnormal samples for training in advance. This is critical in real deployment because it is unrealistic to train the system by providing all kinds of abnormal activities. Another advantage is that our training procedure is an unsupervised learning fashion, which does not need to specify the number of kinds of normal activities. In other words, it is not necessary to label the training samples. This advantage will facilitate the mass deployment in different locations, and it can greatly reduce the human labor spent on training sample preprocessing. We demonstrate the effectiveness of our approach using real data collected from PIR sensors attached to the ceiling of the monitoring region. It shows that, as the number of training samples increases, the performance of our system will improve accordingly.

In the future, we wish to continue in the direction of detecting abnormal activities from continuous data streams. We will investigate how to incorporate the location information with the motion information to improve the performance of our system. A robust and reliable abnormal activity detection system is the most important prerequisite of the home-based assisted living paradigm.

## Figures and Tables

**Table 1:** Confusion matrix.

		Actual Activity	
		Abnormal	Normal
Predicted	Abnormal	True Positive (TP)	False Postivitive (FP)
Label	Normal	False Negative (FN)	True Negative (TN)

**Table 2:** Area under the ROC curve (AUC) values with different algorithms and different numbers of training samples.

	240 Samples	160 Samples	80 Samples
SC+OSVM	0.863	0.433	0.710
SC+iForest	0.375	0.404	0.332
OneHMM	0.354	0.379	0.360
